# Menopause and metabolic syndrome: anthropometric, lipid, and dietary profiles

**DOI:** 10.1590/1806-9282.20231571

**Published:** 2024-07-19

**Authors:** Virginia Barros Sgorlon Gonçalves, Sônia Maria Rolim Rosa Lima

**Affiliations:** 1 Santa Casa de São Paulo School of Medical Sciences, Department of Obstetrics and Gynecology – São Paulo (SP), Brazil.

**Keywords:** Diet, Questionnaire, Metabolic syndrome, Menopause

## Abstract

**OBJECTIVE:**

The aim of this study was to characterize the anthropometric, lipid, and dietary profiles of postmenopausal women with metabolic syndrome attending a public health service and compare them with a group of women without metabolic syndrome.

**METHODS:**

A cross-sectional study was conducted with 60 postmenopausal women who were divided into two groups: control group and metabolic syndrome group, attending the Climacteric Outpatient Clinic at Santa Casa de São Paulo Hospital, Brazil, between February 2019 and December 2021. Participants were evaluated using a validated semi-quantitative food frequency questionnaire, body mass index, waist circumference, and serum laboratory tests.

**RESULTS:**

Significant differences were observed between the groups regarding body mass index and all parameters of metabolic syndrome. The nutritional profile revealed an imbalance in the number of food portions consumed, particularly in the intake of carbohydrates in the form of flour and sweets, which was higher in the metabolic syndrome group.

**CONCLUSION:**

The analysis of the three profiles of postmenopausal women revealed significant imbalances, particularly in the metabolic syndrome group, highlighting the importance of regular adjustments and evaluations during this phase of a woman's life.

## INTRODUCTION

Metabolic syndrome (MetS) is a risk factor for a number of chronic noncommunicable diseases with high levels of mortality and decreased quality of life. Prevalence in women is around 1.5 to 2 times higher than in men^1^.

Metabolic syndrome appears more frequently during the menopausal transition and worsens after menopause. It represents a public health problem, with significant implications for quality of life, healthcare, as well as social and economic aspects^2^.

In clinical practice, the criteria for MetS defined by the US National Cholesterol Education Program Adult Treatment Panel III (ATP III) are widely used for their simplicity and practicality. MetS is diagnosed based on the presence of three out of five factors^3^.

The consumption of a diet rich in saturated fat, starchy carbohydrates, and high daily caloric intake is associated with numerous chronic noncommunicable diseases^4^.

To investigate possible relationships between dietary habits and MetS, we employed anthropometric assessment; conducted routine serum tests for total cholesterol, lipid fractions, triglycerides, and glucose; and applied a validated semi-quantitative food frequency questionnaire (FFQ)^5,6^.

## METHODS

The protocols followed the ethical standards of the Declaration of Helsinki and were approved by the Research Ethics Committee of the Irmandade da Santa Casa de Misericórdia de São Paulo (CEP: 5.322.400). All volunteers recruited signed the informed consent form. Data collection was performed at the moment the patient agreed to participate in the study.

The sample consisted of 60 postmenopausal women aged between 44 and 64 years, who were divided into two groups: MetS group (MetSG, n=30) and control group (CG, n=30). A cross-sectional study was conducted in the public health service with patients attending the Climacteric Outpatient Clinic of Santa Casa de São Paulo Hospital between February 2019 and December 2021.

Inclusion criteria were as follows: postmenopausal women aged up to 64 years, BMI≥18.5 to <35 kg/m^2^, amenorrhea ≥12 months, and FSH≥30 mU/mL. Exclusion criteria were as follows: BMI>35 kg/m^2^, illicit drug use or alcohol abuse, a history of bariatric surgery, cancer, or cardiovascular disease. The diagnosis of MetS was determined according to the ATP III (Adult Treatment Panel) guidelines, widely used for their simplicity and practicality: (1) waist circumference>88 cm; (2) HDL cholesterol concentrations <50 mg/dL; (3) triglycerides ≥150 mg/dL; (4) blood pressure levels ≥130/85 mmHg; and (5) fasting blood glucose ≥110 mg/dL. MetS is diagnosed based on the presence of three out of five factors^3^.

Through a standardized anamnesis, the following were observed: anthropometric profile, in which weight was measured in kilograms (Balmak digital scale, model BK 200, accuracy of 0.1 kg) with the patient in an upright position and minimally dressed, height was measured in centimeters using a vertical stadiometer, and waist circumference (WC) was measured in centimeters at the midpoint between the last rib and the iliac crest using a measuring tape. Body mass index (BMI) was calculated as weight in kilograms divided by the height in square meters (kg/m^2^)^7^. For the nutritional profile, a validated semi-quantitative FFQ was completed in a person, which contains nine frequency options: never or<1 month, 1–3 per month, 1 per month, 2–4 per month, 5–6 per month, 1 per day, 2–3 per day, 4–5 per day, and 6+ per day, with 103 questions and 98 foods^7^. Based on the Food Guide for the Brazilian Population, an average intake of food was found to be 2,000 kcal/day^8^.

In the lipid profile, blood samples were analyzed by enzymatic method using the BT 3000 plus device (Wiener lab®, Rosario, Argentina). The LDL value was calculated and obtained using the Friedewald formula (LDLc= CT-HDLc – TG/5)^9^. Blood pressure was measured using a semi-automatic oscillometer (Omron Hbp-112) while the participant was in a seated position^10^.

### Sample size calculation and statistical analysis

The criterion used was a test power of 80%, and a significance level of 5% was considered adequate for evaluating the frequency of the main study variables. Analyses were performed using SPSS version 25.0 (IBM Corp. 2017, Armonk, NY, USA). To compare qualitative variables, we used the chi-square test and, for quantitative variables, we used the Mann-Whitney nonparametric test and the Student's t-test.

## RESULTS

With respect to the analyzed characteristics, there were significant differences between the groups in terms of weight, BMI, VLDL cholesterol, and clinical parameters for MetS, as shown in Table 1. The most prevalent parameter in this study was WC>88 cm, which was present in 100% of the MetSG participants. The results of the FFQ with 19 most common foods on the Brazilian table revealed significant differences in the consumption frequencies of up to three times a day analyzed between the two groups with regard to vegetables, fruit juice, wheat flour, sugary cereals, cakes, cookies, mono- and polyunsaturated fats, sweets in general, fast food, and added sugar, as described in Table 2.

**Table 1:** Socio-demographic, anthropometric, and lipid profiles and clinical parameters of postmenopausal women in the control group and the metabolic syndrome group (Ambulatório de climatério- FCMSCSP–2022).

Sample characteristics	Control group	MetS group^1^	p-value
Age (years)*	55.0 (±4.9)	56.0 (±5.3)	0.339
Weight (kg)*	64.0 (±10.2)	76.0 (±8.0)	0.000
Height (m)**	1.55 (1.48–1.77)	1.59 (1.44–1.72)	0.239
BMI (kg/m²)**	25.4 (18.8–33.2)	29.7 (24.0–35.0)	0.000
Time after menopause (years)**	4.5 (2.0–16.0)	4.5 (2.0–23.0)	0.922
HDL cholesterol (mg/dL)**	59.0 (102.0–42.0)	50.5 (84.0–34.0)	0.002
LDL cholesterol (mg/dL)**	120.5 (236.0–81.0)	121.0 (161.0–50.2)	0.217
VLDL cholesterol (mg/dL)**	21.5 (40.6–10.0)	32.1 (69.2–17.2)	0.001
Non-HDL cholesterol (mg/dL)**	150.4 (269.6–101.2)	151.2 (201.4–73.6)	0.929
Total cholesterol (mg/dL)**	209.6 (325.6–168.2)	198.3 (249.4–158.0)	0.079
Triglycerides (mg/dL)**	107.5 (203.0–50.0)	158.5 (346.0–15.8)	0.001
Systolic blood pressure (≥130 mmHg)§	12.0 (±1.6)	13.6 (±1.2)	0.024
Diastolic blood pressure (≥85 mmHg)§	7.7 (±1.1)	8.4 (±0.8)	0.024
Waist circumference (>88 cm)§	88.8 (±8.1)	102.0 (±8.7)	0.000
Fasting blood glucose (≥110 mg/dL)§	93.5 (±9.7)	105.6 (±14.0)	0.001
Triglycerides (≥150 mg/dL)§	115.3 (±38.6)	161.7 (±52.9)	0.002
HDL cholesterol (<50 mg/dL)§	63.5 (±14.1)	52.5 (±12.5)	0.002

p<0.05;

* Student's t-test (mean and SD);

** Mann–Whitney test (median, minimum, and maximum);

§ iChi-square test (mean and SD); BMI: body mass index; kg: kilograms; m: meter; kg/m^2^: kilogram per square meter; mg/dL: milligram per deciliter; HDL: high-density lipoprotein; LDL: low-density lipoprotein; VLDL: very-low-density lipoprotein; mmHg: millimeter of mercury; cm: centimeters; MetS^1^: metabolic syndrome; FCMSCSP: Faculdade de Ciências Médicas da Santa Casa de São Paulo. Bold indicates statistically significant p-value.

**Table 2:** Frequency of ingestion of 19 food items from the FFQ, up to three times a day, of postmenopausal women in the control group (n=30) and the metabolic syndrome group (n=30) (Ambulatório de Climatério – FCMSCSP–2022).

Foods	Partial number	Control group	MS^1^ group	Total n=60	Chi square
	nº	%	%	%	p
Assorted meats	11	20.0	16.6	18.3	0.238
Fish	1	0.0	3.3	1.6	0.339
Eggs	18	23.3	36.3	30.0	0.364
Whole animal derivatives	33	40.0	70.0	55.0	0.084
Semi-skimmed and skimmed	11	20.0	16.6	18.3	0.238
Greens and vegetables	22	46.6	26.6	36.6	0.007
Roots	5	6.6	10.0	8.3	0.309
Legumes	38	66.6	60.0	63.3	0.191
Cereals	42	70.0	70.0	70.0	0.366
Fruits	23	50.0	26.6	38.3	0.059
Fruit juice	3	6.6	3.3	5.0	0.009
White and whole wheat flour	31	33.3	70.0	51.6	<0.001
Sugary cereal, cake, cookies, and candy	6	3.3	16.6	10.0	<0.001
Mono- and polyunsaturated fat	11	36.6	0.0	18.3	0.001
Sweets in general	13	16.6	26.6	21.6	<0.001
Fast food	4	0.0	13.3	6.66	0.009
Alcoholic beverage	0	0.0	0.0	0.0	0.079
Regular soda and artificial juice	9	10.0	20.0	60.0	0.079
Sugar for added	25	36.3	46.6	41.6	0.004

p<0.05; Chi-square test (mean and SD); N partial: number of participants who responded positively to the frequency of consumption of the 19 food items; MS^1^: metabolic syndrome group; FFQ: Food Frequency Questionnaire; FCMSCSP: Faculdade de Ciências Médicas da Santa Casa de São Paulo. Bold indicates statistically significant p-value.

## DISCUSSION

To help promote a better understanding of MetS and its possible treatments in postmenopausal women, we analyzed a series of variables that constitute risk factors for chronic noncommunicable diseases, as previous studies have adopted a selective approach, considering the individual's nutritional, anthropometric, and lipid profiles separately^11,12^.

The overweight and obesity in women with MetS were also found in other studies^13-15^. The prevalence of WC >88 cm present in 100% of MetSG can be observed in studies that analyzed the anthropometric profile of participants^13,16^. Regarding changes in the lipid profile, we found similar reports in the literature concerning postmenopausal women with MetS^17,18^.

When we analyzed the nutritional profile of postmenopausal women using the FFQ and examined the consumption of 19 foods up to three times a day, we observed differences between the two groups in relation to the intake of certain nutrients. The MetSG had higher carbohydrate consumption, mainly refined starchy, bakery products, sugary cereals, cakes, sweet biscuits, sweets in general, above the recommended limit (up to 55% of total daily energy intake), and added sugar, above the recommended limit (up to 10% of the total energy ingested), than the CG. The fat intake for this group was also higher than recommended (up to 35% of total daily energy intake), mainly due to consumption of fast food rich in saturated fat and sodium according to the Brazilian Food Guide^8^.

The World Health Organization recommends that sugar consumption be <10% of daily energy intake^19^, while <5% is recommended in the United States and the United Kingdom^20^. Simple sugar intake is associated with increased blood pressure, WC, serum triglyceride, glucose concentrations, and a significantly increased risk of developing MetS^18^.

The CG had a significantly higher consumption of greens and vegetables. According to the results of a meta-analysis, there is a possible relationship between the decrease in fiber intake from vegetables and increased risk factors for MetS^21,22^. The CG also showed higher consumption of items such as good sources of fat like olive oil, compared to the MetSG. These results are similar to other studies^23-25^.

Diet analysis and its impact on anthropometric assessment and laboratory tests are of fundamental importance for planning health promotion interventions and managing comorbidities in postmenopausal women. Adequate nutrition and a healthy lifestyle should be taken into consideration as important factors in this phase of a woman's life^16^.

However, it is important to highlight that the completion of the FFQ regarding the last year can introduce some dispersion in the results, considering that it relies on the participants' good memory and honesty during completion, which may compromise the analysis performed.

Therefore, we hope that our study opens new horizons for further research and scientific investigations in this field.

## CONCLUSION

The analysis of the anthropometric, lipid, and dietary profiles of postmenopausal women with and without MetS revealed an imbalance in macronutrient intake and portion sizes consumed. The MetSG had a higher prevalence of physical and laboratory alterations compared to the CG, which exhibited profiles closer to the ideal.

Therefore, there is a need to emphasize the importance of nutritional adjustments and regular evaluations during this phase of life in order to minimize the risks of health complications associated with this condition.
